# Evolutionary emergence of collective intelligence in large groups of students

**DOI:** 10.3389/fpsyg.2022.848048

**Published:** 2022-11-02

**Authors:** Santos Orejudo, Jacobo Cano-Escoriaza, Ana Belén Cebollero-Salinas, Pablo Bautista, Jesús Clemente-Gallardo, Alejandro Rivero, Pilar Rivero, Alfonso Tarancón

**Affiliations:** ^1^Department of Psychology and Sociology, University of Zaragoza, Zaragoza, Spain; ^2^Department of Sciences of Education, University of Zaragoza, Zaragoza, Spain; ^3^Department of Theoretical Physics, University of Zaragoza, Zaragoza, Spain; ^4^Institute for Biocomputation and Physics of Complex Systems, University of Zaragoza, Zaragoza, Spain; ^5^Kampal Data Solutions, Zaragoza, Spain; ^6^Department of Specific Didactics, University of Zaragoza, Zaragoza, Spain

**Keywords:** collective intelligence, social experiment, web platform, mathematics test, moral reasoning

## Abstract

The emergence of collective intelligence has been studied in much greater detail in small groups than in larger ones. Nevertheless, in groups of several hundreds or thousands of members, it is well-known that the social environment exerts a considerable influence on individual behavior. A few recent papers have dealt with some aspects of large group situations, but have not provided an in-depth analysis of the role of interactions among the members of a group in the creation of ideas, as well as the group’s overall performance. In this study, we report an experiment where a large set of individuals, i.e., 789 high-school students, cooperated online in real time to solve two different examinations on a specifically designed platform (Thinkhub). Our goal of this paper 6 to describe the specific mechanisms of idea creation we were able to observe and to measure the group’s performance as a whole. When we deal with communication networks featuring a large number of interacting entities, it seems natural to model the set as a complex system by resorting to the tools of statistical mechanics. Our experiment shows how an interaction in small groups that increase in size over several phases, leading to a final phase where the students are confronted with the most popular answers of the previous phases, is capable of producing high-quality answers to all examination questions, whereby the last phase plays a crucial role. Our experiment likewise shows that a group’s performance in such a task progresses in a linear manner in parallel with the size of the group. Finally, we show that the controlled interaction and dynamics foreseen in the system can reduce the spread of “fake news” within the group.

## Introduction

The measurement of individual intelligence has been possible since the beginning of the 20th century (Spearman, 1904). Around 2010, an accurate experiment was developed with the purpose of testing the concept of collective intelligence as generated by a group of individuals. Researchers from Carnegie Mellon University and MIT published an article in Science that demonstrated the existence of a statistical parameter that allowed to predict the best or worst performance of a group of people in solving tasks of different types (Woolley et al., 2010). More recent papers by the same authors have reported similar results with other types of tasks and online frameworks (Engel et al., 2014; Hjertø and Paulsen, 2016; Meslec et al., 2016; Woolley and Aggarwal, 2020).

That body of investigation has led researchers to identify a series of relevant variables capable of explaining the emergence of those results. In total, two different levels of analysis have been considered (Woolley and Aggarwal, 2020): on the one hand, top-down processes, i.e., interaction processes that emerge within the group, and, on the other hand, bottom-down processes, which depend on the characteristics of the group’s components. Certain studies have thus focused on the characteristics of the members of the group by taking relevant variables into account, such as the gender of participants (Curşeu et al., 2015), cognitive diversity (Aggarwal et al., 2019), emotional intelligence (Hjertø and Paulsen, 2016), and social sensitivity (Woolley and Aggarwal, 2020). Nonetheless, hypotheses assuming a more pronounced emergence of collective intelligence in groups with a higher proportion of women (who have higher levels of social sensitivity and cognitive diversity) did not find support in results from other research teams such as Bates and Gupta (2017), where high levels of individual intelligence proved to be the best predictors of a group’s performance.

The analysis of top-down factors has focused on the interaction among the members of a group, analyzing several aspects such as team creativity, group heterogeneity, individual incentives, consensus-seeking, duration and continuity of the interaction, and the successive order of turns taken by the group’s members (De Vincenzo et al., 2017; Aggarwal and Woolley, 2018; Bernstein et al., 2018; Dai et al., 2020). This line of research tends to focus on situations with small groups (between 2 and 5 individuals), which makes it difficult to study how collective intelligence behaves according to group size, particularly in the case of large groups. Such studies either tend to favor face-to-face interactions or online experiments featuring a similar number of participants Engel et al. (2014). Nonetheless, the Internet and its unprecedented possibilities of interaction offer a new field for the study of collective intelligence. Recent tools make it possible to design experiments of variable length, with a much larger set of participants, and featuring different types of tasks and problems. A new concept similar to collective intelligence has been introduced in this context: crowd intelligence, i.e., the capacity of a group to solve complex tasks. This concept, however, tends to be poorly defined (Bigham et al., 2018).

Group size is now gaining importance as a crucial factor for the analysis of the emergence of collective intelligence in group interaction. Mao et al. (2016) point out the need of investigating the behavior of such large groups in experiments featuring a limited number of variables, and taking place in natural contexts that reflects the complexity of real-life situations. Analyses have thus been conducted on simulations that study a group’s degree of heterogeneity (Dai et al., 2020), as well as a group’s tendency to consensus (De Vincenzo et al., 2017; Massari et al., 2019) the social learning and the group size (Garg et al., 2022). In experimental situations and natural contexts, Tinati et al. (2014) studied the problem of collaborative creation in their analysis of citizen science projects and the different levels of involvement of volunteers. In an experimental context featuring smaller groups, Ali and Shah (2007) proposed a framework based on Lagrangian particle dynamics for the segmentation of high-density crowd flows and the detection of flow instabilities; moreover, Mehran et al. (2009) applied the social force model to predict anomalous behavior patterns in crowd videos. Controlled experiments are also a good option to investigate collective intelligence in large groups, being the work developed by Toyokawa et al. (2019), a good example of those experiments.

These are some of the difficulties encountered in studies that have attempted to verify how large groups of up to thirty people should collaborate together to carry out a task (Toyokawa et al., 2019). The social learning strategies that emerge during the process become the key factors in the achievement of agreement within the group, and they lead to a higher quality solution than individual ones. Further behaviors appear, such as the herd effect: i.e., an initial tendency to conformity that limits the group’s creativity, or a wide dispersion of alternatives that need to be unified and coordinated (Mao et al., 2016; Aggarwal and Woolley, 2018; Toyokawa et al., 2019). Further difficulties are the decrease in creativity because group members copy one another (Lorenz et al., 2011), and the excessive influence of leaders (Iyengard et al., 2011; Mann and Helbing, 2017; Fontanari, 2018). To take advantage of a collective effort to tackle problems through the process of exchanging ideas, cross-fertilization requires the presence of a system that exercises the role of “facilitator,” i.e., of control and management of the group’s collective work (Mann and Helbing, 2017; Aggarwal and Woolley, 2018; Gimpel et al., 2020).

The type of interaction between the members and the type of demand or task that is posed are essential to achieve an understanding of emerging behaviors. For example, tasks that are more open generate a greater dispersion of responses and a reduction in the number of copies, which are more frequent in tasks that are more closed (Toyokawa et al., 2019). Also, the type of interaction or the prestige of certain participants can exert an influence on copying behaviors. For example, Bernstein et al. (2018) found that an intermittent interaction model, as opposed to an individual work model, improves creativity as well as the quality of responses to tasks that are poorly defined. Also, Lorenz et al. (2011) verified how social prestige acts as a convergence mechanism, but without implying an improvement in response quality.

In conclusion, the study of collective intelligence in large groups is a complicated task due to the large number of variables involved, as we have already seen when reviewing the levels top-down and bottom-up. The complexity will even increase when resorting to online environments to facilitate interaction in large groups (Pescetelli et al., 2021), with the emergence of different models of interaction, combining tasks of an individual nature or those of small and large groups (Navajas et al., 2018; Yahosseini and Moussaïd, 2020) or even the mechanism of assignment of the participants to the groups (Almaatouq et al., 2020; Pescetelli et al., 2021). A final contextual variable that is hardly taken into consideration in these studies with large groups is the individual differences between the participants, who may take different strategies to solve the task and thereby generate different dynamics (Hertz et al., 2021). Apart from the nature of the interaction, the type and design of the tasks proposed in the collective intelligence experiments is essential. Thus, for example, the response format can vary, and thus, compared to the classic collaborative tasks carried out by a group (Woolley et al., 2010), in online tasks, it is possible to develop individual solutions of the task and then add elements of collaborative work (Almaatouq et al., 2020; Toyokawa and Gaissmaier, 2022). Likewise, on these individual solutions, it is possible to find environments oriented toward the search for consensus or more open in which a high heterogeneity of responses can be maintained. Finally, the type of tasks proposed can also allow a great diversity of answers, from completely open tasks for which it is difficult to establish a quality assessment to more closed tasks with correct and incorrect answers and in which it is possible to easily generate elements of group feedback that can allow the emergence of new group mechanisms (Almaatouq et al., 2020; Pescetelli et al., 2021).

This complexity has led some authors to state that the emergence of collective intelligence in large groups may ultimately depend on contextual factors and specific dynamics that emerge in the interaction (Almaatouq et al., 2020; Sulik et al., 2022), since, although a group can generate solutions to poorly defined problems, it can also lead to the maintenance and perpetuation of non-optimal solutions (Toyokawa and Gaissmaier, 2022). Thus, it seems necessary to investigate and know under what conditions this collective intelligence can be generated. Aiming to test the possibility of generating collective intelligence in an online collaborative environment designed for large groups, researchers from the Institute for Biocomputation and Physics of Complex Systems of the University of Zaragoza^1^ and the Kampal Data Solutions firm^2^ have designed the Thinkhub platform.^3^ Its goal is to generate an interaction framework with the purpose of fostering the emergence of high-quality solutions to problems presented to a group: i.e., the emergence of collective intelligence. Aiming to achieve an orderly discussion climate and to produce ideas that are globally correct, our platform design takes into account the potential dangers involved in the interaction within large crowds. Such dangers are as follows:

• Noise: the profusion of unfiltered ideas creates confusion and makes it difficult to carry out reflective, personal work.
• Disruption: In the group, there is a percentage of people who do not seek to generate solutions but, rather, confusion or fake news.
• Influencer weight: An opinion is most highly regarded if proposed by people with social influence, independently of its validity.

Furthermore, individual behavior within the group presents difficulties that hinder the achievement of constructive participation on the part of a large percentage of participants, mainly due to the following factors:

• Isolationism: Many people choose to disconnect from the environment, either by their own decision, or because they feel different or ideologically detached from it.
• Dispersion: There is a tendency to produce as many solutions as there are people present.
• Leadership syndrome: A certain percentage of people prefer to lead or participate in a small group with their own ideas, rather than adhere to better ideas that are prevalent in a different (generally larger) group.

As an alternative to these problems, we used an online platform that allowed us to parameterize the number of interactions between persons, starting from zero, then with four neighbors, and finally achieving a global view. It is important to point out that five individuals are the maximum size of groups featured in traditional, face-to-face collective intelligence studies (Woolley et al., 2010) and in certain online replications (Engel et al., 2014; Meslec et al., 2016; Bates and Gupta, 2017). On our platform, however, by interacting with four neighbors, participants can analyze their neighbors’ information, make assessments, and choose either to edit/modify their own answer or to copy one of their neighbors’ answers. In the final phases 5–6, the system confronts participants with a greater number of responses, while also incorporating the social prestige criterion: the responses displayed by the system are the most popular ones and involve processing a greater amount of information, since the latter does not stem from four neighbors alone, but from the entire group.

Thus, the aim of this paper was to report the results of an experiment with a large group of students (*n* = 789) who were confronted with a series of mathematical and social reasoning tasks, similar to the ones that have appeared in other collective intelligence experiments. Our hypotheses are that the participants will remain active throughout the experiment, but their activity will depend on the conditions of the interaction into the network. On the other hand, the participants will carry out two different tasks, different mathematical and social reasoning tasks. Both are designed with an open task format, with different questions, one of which is designed in an answer format that can act as a fake news to check what happens with information that could respond to this format.

## Materials and methods

### Participants

Our experiment was carried out on a total of 900 students, with the active participation of 789 students, 16/17 years old, enrolled in 33 schools in Aragon (Spain). The students were following a non-compulsory 2-year course (in Spain, compulsory education finishes at 16), aimed to help students prepare for entrance to university. The students had to solve two tasks in the form of exams: first, a moral dilemma (MD), then a mathematics test (MA). Each examination lasted around 45 min, plus several initial minutes required to synchronize with the platform. All students had a computer with an Internet connection. A previous test—on two other topics, History and Physics—ran for a week to allow students to familiarize themselves with the platform. The experiment was anonymous: we know to which school each student pertained, but we have no further data about them. The participants’ position in the platform network was randomly assigned; therefore, they did not know their neighbors’ identity.

The schools participating in the experiments responded to a call from the Education Department of the Regional Government of Aragon, which featured those experiments as an official activity. Prior to completion, families were informed *via* a letter about the study’s purpose and procedure and about the guaranteed anonymity of all participants. In the same letter, students were informed of their voluntary participation, and that they could refrain from doing so if their families did not agree or if they themselves did not wish to take part. Thus, only students willing to participate in the experiment received a code to access the platform. Subjects received no compensation for participating in the study. This study was carried out in accordance with the recommendations of the Council of the British Educational Research Association in the second edition of their Ethical Guidelines for Educational Research (2011). Compliance with the standards of the Declaration of Helsinki on human experimentation was guaranteed at all times. Furthermore, the full research program containing this and further experiments to be carried out in the near future was approved and validated by the Committee of Ethics in Research of the Government of Aragon.

### Model

**FIGURE 1:**
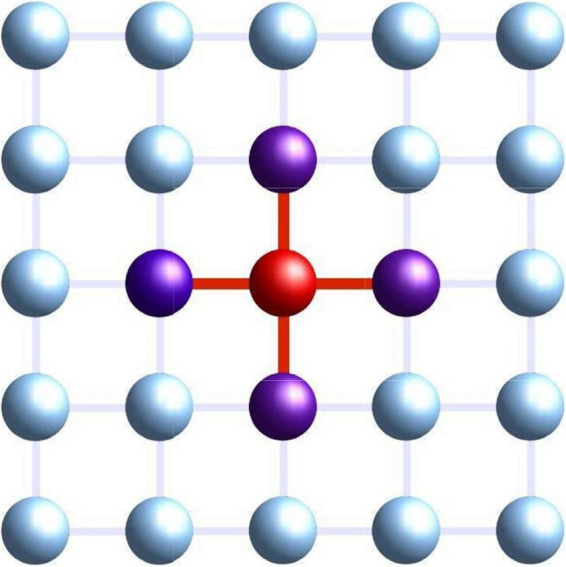
Geometric virtual disposition of students. For instance, the red one is connected with (can see or copy from) four neighbors. Used with permission from Kampal Data Solutions.

**FIGURE 2:**
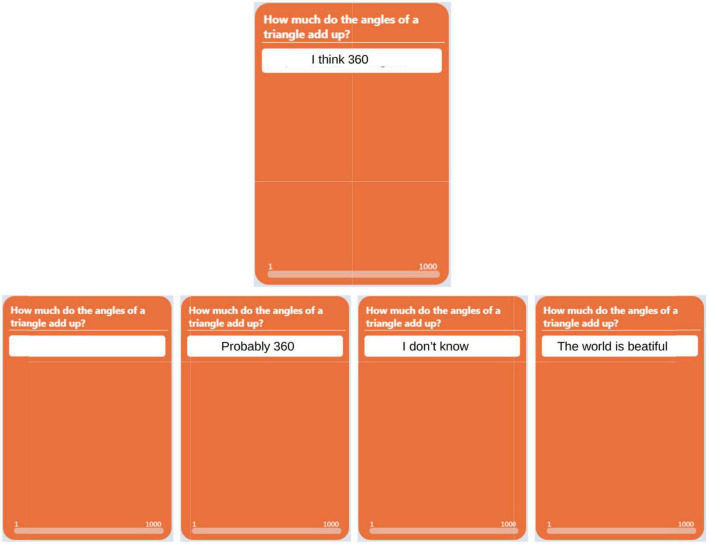
Scheme of online platform view (Phase 2) from the viewpoint of one student (upper position). He/she can see his/her four neighbors (lower position). Used with permission from Kampal Data Solutions.

The experiment creates a virtual online environment designed to host a large set of people who collaborate to solve two tasks. The platform considers each individual (rather, his/her answers to the examinations) as a node in a 2D lattice. Individuals can work on their solutions, and in certain phases of the experiment, they can see responses from neighbors in the lattice, or they can copy from them. We can picture this process as an interaction within a physical magnetic system where a given node of a lattice will see its magnetic state modified by the state of its neighbors. Indeed, our online model was initially based on magnetic models such as the Ising Model, where elementary nodes (atoms or individuals) are located on the vertex of a square lattice, and a node only interacts with its immediate neighbors. In Figure 1, we can see a 5 × 5 lattice where we show in purple the neighbors of a (red) node. In our experiment, lattice size was around 30 × 30 nodes, i.e., 30 × 30 participating subjects. In magnetic models, interaction is generated by electromagnetic forces (between neighbors only) which change the state of the nodes; in our human lattice, interaction appears when a node can see or copy neighbor solutions (refer to Figure 2 for a graphical representation of the online platform used in the experiment).

Our experiment was divided into 6 effective phases (refer to Table 1); we started from strictly local interactions (where students could access the solutions of only four neighbors) and progressed with time to more global interactions (where they could access the most popular solutions stemming from the entire group). In the course of those phases, we introduced several mechanisms based on the ideas borrowed from statistical mechanics and biological evolution. These mechanisms were designed to minimize problems observed in large group interactions, such as those mentioned above. Each node interacts with its four neighbors, which also interact with theirs, thus allowing for effective, long-range interaction. The different ideas generated by the participants are seen, copied, or discarded by other participants. In the course of the system’s evolution, those ideas will likewise evolve. Hence, we associate a quantitative notion of “biological health” to each idea as a function of the number of times it has been copied. At a certain point, we impose the extinction of those ideas, which have not been sufficiently copied, as a way of obliging the group to reach a consensus. Let us briefly present this process in further detail.

**TABLE 1:** Phases of the experiment and basic properties of each phase.

Phase	Edit items	Visual and copy scope	Dynamical items delete
1	Yes	No	No
2	Yes	Frozen 4 neighbors	No
3	Yes	Real-time 4 neighbors	No
4	Yes	Real-time 4 neighbors	Yes
5	Yes	Top10 solutions	Yes
6	No	Top10 solutions	No

The general operation is the following: Each user owns their own solutions and can create or modify them. The only potential interaction they can have with other users is to view their solutions or copy from them. Indeed, users can copy Items from their immediately accessible neighbors. In this way, even with interaction restricted to a local level, information spreads across the entire lattice if items are copied by successive users. This is similar to coherence phenomena and phase transition in magnetic systems (McCoy and Wu, 2014), where global propagation is likewise possible. During the first three phases, we enable this interaction progressively. In Phase 1, participants are not yet allowed to see any other solution. In Phase 2, they can see and copy the solutions that their neighbors have proposed at the end of Phase 1. In Phase 3, visible solutions are continuously updated each time that any neighbor presses the “save” button. We maintain the system’s local interaction geometry up to Phase 5. When Phase 5 starts, we move to global geometry (non-local interaction in physical language), and users are shown the best 10 solutions (Top10), defined as solutions with the largest biological health (refer to the preceding paragraph). Users can copy from Items in the Top10 to their own solution, and they can still propose new solutions. In Phase 6, the final phase, users can no longer edit their solutions, but only can copy from existing solutions from the Top10.

We chose to design the system’s geometry as a square with periodic boundary conditions. In this way, we start (after the first phase) with four neighbors. Different geometries could likewise have been applied: for instance, a tridimensional lattice (six neighbors), a four-dimensional one (eight neighbors), etc. Even a general graph connection is possible, with varying distributions of connectivity (Sethi et al., 2019). Having a reduced number of neighbors favors the visibility of other users; this, in turn, helps to reduce noise. In accordance with our vision of the system as a statistical model, our main objective was to ensure the propagation of ideas at all levels, eventually across the whole lattice, similar to a magnetic model that has reached the critical point (Amit and Martin-Mayor, 2005). For any possible number of neighbors, or different geometries, we in fact have different modes of idea propagation. Speed and overall times may change, but it is well-established that collective properties, such as the global propagation of information across a system, do not depend on such details. According to the Universality Hypothesis in Statistical Mechanics (Amit and Martin-Mayor, 2005), different geometrical designs exert an effect on overall times, but they do not alter interactions, propagation, interchange, participation, etc. Our choice was thus to opt for a reduced number of neighbors to minimize dispersion and to stimulate reflective work.

Let us describe these phases more specifically. Our objective in Phase 1 is to provoke a necessary, isolated period of serious, profound individual reflection. In Phases 2–3–4, the number of participants in each interaction is five: i.e., the central node and its four neighbors. In these latter phases, we observe two effects:

• First, social influence, one of the factors that tend to affect responses in this type of tasks (Lorenz et al., 2011; Bernstein et al., 2018);
• Second, a greater amount of information becomes available for the participating subject to assess. This amount of information implies a greater diversity of options, which, in turn, increases cognitive diversity, another key factor in the emergence of collective intelligence (Aggarwal et al., 2019).

From the user’s point of view, the system always works in the same way. He/she has constant access to the task questions, as to the different interaction mechanisms (neighbors’ answers); moreover, the same amount of time is available in each phase. In Phase 1, the students work on the tasks individually, without interacting with their neighbors. In the course of the following three phases, students interact with their four nearest neighbors, and they are allowed to view their neighbors’ answers to the task, as well as to copy and/or subsequently modify them. In this way, an idea generated in the first phase can travel through the lattice if the other students find it interesting, since the process allows for effective interaction between distant nodes. Our research team then ranks ideas according to their popularity, i.e., the number of times they have been copied by other students. In Phase 4, less popular ideas are deleted from the system. As a reflection of phenomena observed in biological evolution, we call this process the extinction or deletion phase.

During the final two phases (Phases 5 and 6), the students only have access to the Top10 solutions that were most frequently copied in the preceding phases. This process aims to force the group to reach a consensus and produce one sole answer to the questions. Notice that the platform, in this way, is acting as the facilitator to whom we referred in the introduction. But the facilitator is created here by the group itself, without any external intervention. In this sense, we can claim that the resulting answers can be regarded as the group’s original creations, without external influences.

We need a series of objective criteria to categorize and classify responses, since they have only been generated from internal data, without external control. This is an important constraint, as the system should evolve solely thanks to its internal dynamics and without any external interference if we want the created solution to be ascribed to the group’s authorship alone. The quality criterion we are applying here is the number of copies, and we define the (time-dependent) Frequency of an Item as the number of actual copies thereof in the system at that moment. We will use this item frequency as a measure of biological health for the evolutionary algorithms.

We assume that within this model, the system’s evolution must converge toward a solution that lies close to the correct one. The primary objective of this experiment is to ascertain whether this hypothesis is correct. Moreover, as users can generate false (or fake) answers as well as out-of-context remarks (jokes, political statements, insults, etc., which we refer to below as troll answers), we also want to test the system’s resilience with respect to such phenomena. The entire system is based exclusively on user solutions, without external influence. Furthermore, an important aspect is the absence of a like option to vote or promote other users’ Items. Therefore, to support a solution, the user must adopt it as their own by copying it, thereby increasing the Frequency (biological health) of the copied Item, which they adopt as their own.

In our experiment, troll answers generated by the users did indeed appear, and it was obvious that they had been deliberately created; therefore, we tested our system to see how it withstood this challenge. The generation of fake news is not natural in such a controlled academic experiment. To ensure that the system remained autonomous, we introduced trick questions designed to mislead students who have a tendency to look for solutions on the Internet: they find a solution which they assume is correct, although actually, it is not. In this way, we forced the system to generate internally fake news that was correct from the users’ point of view, even from that of the answer’s creators (in a similar way as occurs to all the users in real world, except for the fake news item’s originator). This is significant in view of the important differences between fake news and malicious actors (Sethi et al., 2019).

### Dynamics

In a series of pilot tests prior to the main experiment, we observed that individuals do not like to copy other users’ answers. This led to a large set of solutions; in other words, the classification according to biological health became exceedingly large, almost uniform. As an attempt to solve this dispersion problem, we introduced dynamic behavior based on statistical and evolutionary algorithms, to decrease the total number of ideas and stimulate the appearance of a global best solution. The purpose was to simulate the extinction of biological systems to obtain the fittest individuals and encourage mutations. This was implemented in the following manner:

In Phases 4 and 5, we activate an items deletion phase. Consider, for instance, the Item for the first Question Q. At time T, we have, for instance, N_*Q*_ answers, which correspond to M_*Q*_ ≤ N_*Q*_ different Items, each with a frequency F_*i*_ *i* = 1, …, M. We then order these M_*Q*_ answers by Item and by Frequency (or biological health). Our goal is to create an answer-deletion algorithm that will lead to ca. 10 different responses by the end of the experiment (the Top10 set). To achieve this, we establish a linear evolution between (T, M_*Q*_) and (T_*final*_, 10). Then, at time T + dT, we compute the (usually lower) number of items on the line, and we call this number M_*new*_. Then, we delete all items with lower frequencies, only leaving M_*new*_ items for the questions at time T + dT. Users whose answers have been removed see blank items after the deletion. Note that deletion of Item Q generally implies the deletion of not one, but F_*Q*_ responses provided by F_*Q*_ users. The algorithm continues to be applied until, at T_*final*_, only 10 different answers remain for each question.

Finally, in Phase 6, we stop editing and deleting; users can only copy from the Top10 set to their own solutions. In Phases 5 and 6, the Top10 visibility emulates social influence, as solutions are now being viewed by the users as the most popular ones in the system. In MD, the process had an unexpected effect: when extinction stopped, a slightly undesirable answer resulting from troll activity could be observed. Nonetheless, the effect was short-lived.

From a theoretical point of view, to model crowd behavior according to Lagrangian particle dynamics (Ali and Shah, 2007), we previously studied a model with particles in a grid. In our approach, the dynamic is directly applied to user activity: we modify their responses or positions, even deleting certain ones. We modify the real dynamics: this is not a theoretical simulation. Our dynamics are applied directly in the course of the process as actions performed directly upon real users (up until now, we are not foreseeing a modelization of our experiments). The final objective is the global propagation and acceptance of ideas created by the group. Our dynamics work in favor of this goal by modifying interaction, deleting less popular ideas, etc. As we shall see below, these mechanisms enhance the probability of collaboration: for a large number of participants, they ensure that the probability of the propagation of good solutions will be very high.

### Questions and grading mechanism

To test the platform in different contexts, we assessed the application of two problems lying at two opposite methodological poles: a moral dilemma (MD), on the one hand, and a mathematics examination (MA) on the other hand. Apart from using the frequency of copies of answers as a criterion that classifies them according to their biological health, it was necessary to grade the answers to be able to estimate their quality and evaluate the effectiveness of group interaction.

In the Moral Dilemma (MD), we proposed three questions regarding a sexting case. A girl takes an intimate photo of herself and sends it to her boyfriend, who in turn sends it to a friend who makes it public. Students were questioned regarding the three young people’s actions. To carry out a CI measurement, we applied scoring according to the Kohlberg theory of morals (1976, 1989), described as a universal sequence in three stages: preconventional, conventional, and postconventional, featuring two additional substages in each one of the three stages. Each stage is associated with a consideration of what is good, with reasons to act in a moral sense, and with a social perspective, whereby cognition is viewed as the engine of moral development. Kohlberg’s theory provides a frame of reference for the understanding of moral development and of the mechanisms that can promote its progression. Such mechanisms include interaction with other people, along with a system of identification of the mechanisms of moral reasoning *via* dilemmas such as the one presented here (Jespersen et al., 2013; Zhang et al., 2013).

On the other hand, the mathematics examination (MA) consisted of five questions of increasing complexity, from “What is the sum of the angles of a triangle,” to trigonometry items, finishing with a linear equation. Those problems had only one solution and were therefore simpler to grade. The first question was trivial; it was included to ascertain whether students had understood the game, as well as whether they were willing to contribute correctly to the experiment. It served as a probe for the experiment’s validity.

The most complex question in MA was the fifth one, which required the solving of a linear equation to obtain a person’s age. The question was quite similar to the classic “Diophantus equation,” which students could identify and search for on the Internet (as they were allowed to access the Internet in the course of the examination), but the data presented in the equation were slightly different, and the correct solution (56 years old) was not the one found on the Internet (84 years old). Therefore, students who might guess that the problem was the original Diophantus one and who would extract the result from the Internet would be injecting a false solution into the system, mistakenly assuming that it was the correct one. In this way, we could generate a fake answer, and we could study its evolution in a simple way.

To grade the Questions, we proceeded as detailed below. Due to the large number of students, we ran automatic algorithms to grade the answers in the following manner:

• To grade the moral dilemma, our team initially selected 100 random users and considered their answers to the three questions (this represented a total of 552 answers). Those answers were manually analyzed using Kohlberg’s theory (1976, 1989) and a set of keywords was created from them. Using those keywords, we were able to create an algorithm for automatic analysis of the complete set of answers. In this process, we detected the presence of keywords that either denoted typical responses to the levels of moral development established by Kohlberg or could be associated with troll responses unrelated to the task. Following this system, we assigned 0 points to troll answers, five points to answers that contained the keywords of Level 3 of Kohlberg’s moral reasoning, and 8 or 10 points to those answers that contained keywords of Levels 4 and 5, respectively. After applying this rule, we coded the responses according to length, assuming that a greater degree of moral reasoning required a more extended response. This system, without being perfect, was validated based on the 552 random participant responses (Spearman’s Rho = 0.504).
• To grade MA, we established a series of regular expressions matching the digits of the numerical quantities expected to be included in the solution. While this would exclude the answers whose results were given as words, an inspection of the final results showed that it covered most of the correct answers. All items were scored in the range [0,10].

## Results

Let us analyze the results of the experiment from several perspectives, starting with the most general ones and proceeding to the most concrete ones.

### General aspects of idea dynamics

We can create several parameters to monitor the system’s behavior. In the following, we refer to each elementary answer as an Item. A Question is made up of several Items, and a Solution, of several Questions. First, we can consider the total number of Items which the students have introduced in the platform. Notice that if the answer is repeated or copied by other students, the Total Ideas number remains unchanged. Nonetheless, it is cumulative along time. We can also consider the Current Ideas, i.e., the number of different ideas in the system at a given time T. Obviously, for any time T, Total Ideas ≥ Current Ideas, i.e., the difference produced by items deleted or overwritten by the users or by the system dynamics. Total Copies stands for the cumulative number of copies as a function of T. Finally, Deleted Items correspond to the number of (different) Items deleted, counted cumulatively. Remember that the deletion of one item can produce a large number of response (Item) deletions.

**FIGURE 3:**
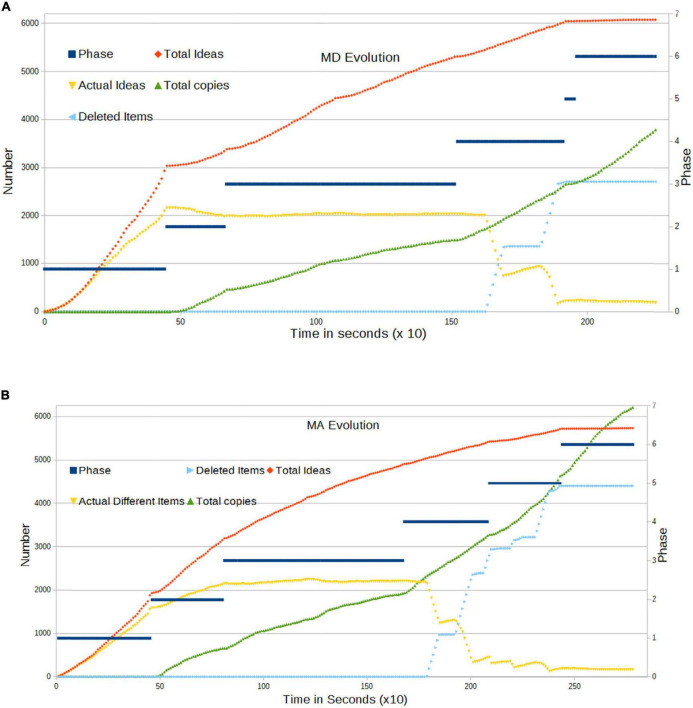
**(A)** Evolution of activity in the MD examination. The dark blue line shows the phase at each moment (right Y axis). Students’ activity corresponds to the red, yellow, and green lines. The light blue line stands for the items deleted for purposes of dynamics. The time unit is 10 s. **(B)** Evolution of activity in the MA examination. The dark blue line shows the phase at each moment (right Y axis). Students’ activity corresponds to the red, yellow, and green lines. The light blue line stands for the items deleted for purposes of dynamics. The time unit is 10 s.

The evolution of these parameters in the course of the two examinations is presented in Figures 3A,B below. The dark blue line serves as a guide to view the phase at each given moment (scale on the right). Based on this, we can describe the experiment from viewpoint of the dynamics of ideas on the platform:

• In Phase 1, we observe a large rate of creation of ideas, even stronger than a linear rate. New ideas appear continuously, starting immediately after *T* = 0. Current Ideas grow more slowly due to the deletion of certain items by users. Notice that, by dint of construction, the number of Current Ideas is limited by the number of allowed items times the number of effective participants.
• When in Phase 2, students start to see and copy the Items from their neighbors, and we observe an important change: the number of Current Ideas stabilizes. This occurs despite the fact that a large number of students continue to create new ideas; however, another important proportion of the population starts to copy from neighbors. The act of copying implies the removal of old ideas; thus, a large number of ideas start to disappear from the system. That number compensates for the new ideas that are being injected.
• In Phase 3, students can see their neighbors’ solution and the changes their neighbors apply in real time. The number of new ideas is now almost constant. Copies compensate for the absence of new ideas.
• In Phase 4, we introduce an evolutionary algorithm *via* Item deletion. The total number of Current Ideas suddenly collapses, and the slope of copies becomes steeper in the correct direction, as students are adopting their neighbors’ ideas. Still, the generation of new ideas continues, but with a lower rate of increase. All this goes in the correct direction of reducing dispersion and converging toward a global solution.
• In Phase 5, students start to see only solutions from the Top10 set, and we do not observe significant differences in these quantities. However, we note the important changes in terms of grades/scores (refer to Section “Evolution of single questions”).
• Finally, in Phase 6, students cannot edit their own answer, and extinction is halted. Current Items now remain almost constant, because it is no longer possible to create new answers.

### Evolution of single questions

**FIGURE 4:**
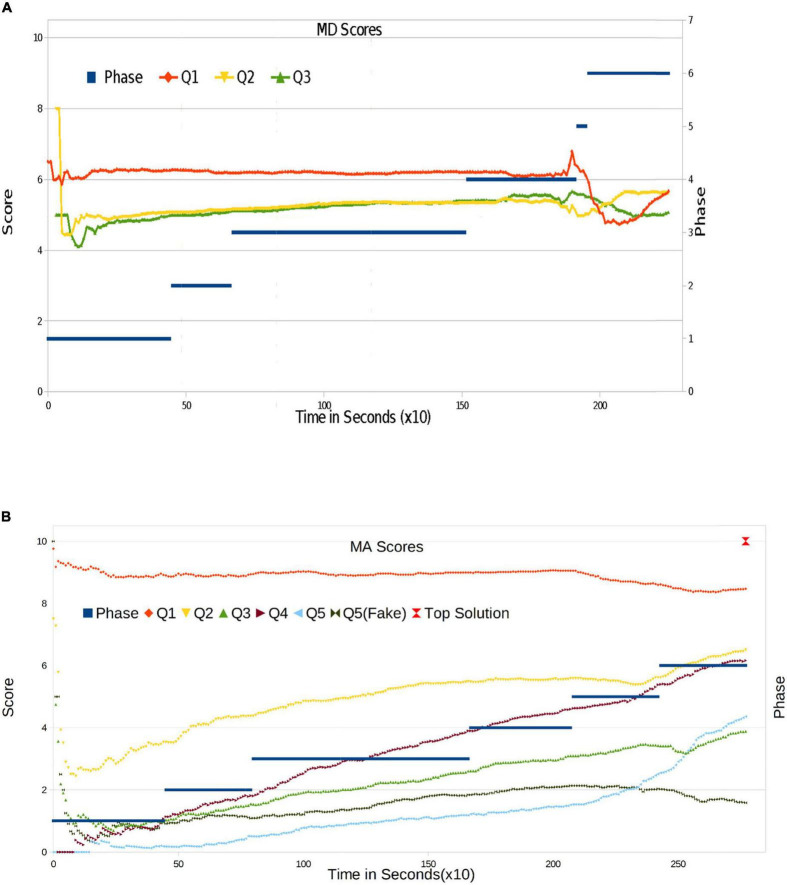
**(A)** Individual question score evolution for MD. The maximum score is 10. **(B)** Individual question score evolution for MA experiment. The maximum score is 10.

We now start to analyze the progression of answer quality by computing the evolution of scores for each question in the exams. Our grading mechanism is described in detail in the Methods Section. We compute the average grade for every question at each moment by taking into account all answers that are not empty (Null). In other words, we only take into account the grades of the actual answers given. The evolution of those grades is shown in Figure 4A (for MD), Figure 4B (for MA).

In MD, the score evolves smoothly, with fluctuations at the onset when there are still few responses. In Q1, we can see a slight decrease toward the end. In the case of Q2 and Q3, evolution is positive up to Phase 6: Q2 evolves positively and Q3 decreases smoothly. The most striking result can be observed in Phase 1, where students are providing answers on their own, and some high-quality responses appear. They probably come from students who have been faced with this type of situation and can provide a more profound reasoning. When answers are added up with one another, this effect disappears and the score tends to stabilize. Large variations do not appear until the end of phase 4. As a consequence of the deletion process, the score falls and is not recovered. In contrast, Q1, after having reached a peak, recovers its score after the deletion phase has begun. This difference could be due to the fact that the participants have elaborated their reasoning in different ways depending on the information contained in each question. Now, they have to analyze more information than before: instead of four neighbors, they only have access to the top 10 answers. The difference in response patterns among the three questions could be due to the fact that students prioritized their response to the first one.

In MA, the behavior of Q1 is similar to Q1 in MD. Let us recall that in MA, the first question (Q1) is trivial: it is designed to test whether students understand the platform and the rules and to see whether they are willing to participate in a constructive manner. We once more observe flat evolution and a slight decrease at the end. In any case, the score for Q1 is the highest one. After extinction dynamics set in, the score has almost reached the maximum (10), followed by a strong decrease, then another growth phase when the deletion rate increases.

The more complex items Q2 to Q5 evolve in a very different way. At the onset, only the very best students are responding. Moreover, they are generally responding correctly, except in the case of Q5. Other students rapidly start to respond; by the end of Phase 1, 80% of questions have been answered, and the initial effect disappears. Q2 starts to grow at a constant rate. For the more difficult questions (Q3, Q4, and Q5), the scores have already stabilized before the onset of Phase 2. In Phase 2, Q2-Q5 show acceleration, with slope change, and they also evolve continuously in Phase 3. Massive deletion takes place in the middle of Phase 4. At this moment, a remarkable change appears, with almost what could be termed as a discontinuity in average scores. Using a physical simile, we could say that the system undergoes a sociological phase transition. Indeed, this is the first relevant result of our experiment: massive deletion obliges users to consider other solutions and also to think about new proposals; because of the knowledge acquired in the meantime, new ideas are now better than the previous ones. At the same time, from Phase 5, the students are allowed access to the group’s Top10 solutions. This also appears to exert a strong influence on answer quality: while in the first 4 phases of MA, the improvement of answer quality was linear along time (and hence in parallel with the increasing size of the group each student is interacting with). Once the students had access to the Top10 solutions (i.e., a representation of the average answer of the whole group), answer quality improved suddenly and dramatically.

It is important to recall that the evolutionary algorithm (refer to Methods Section) can also extinguish good solutions, since the system does not take answer quality into account, only the number of copies. Nonetheless, in this way, we ensure that a purging process takes place, allowing for correct responses to have more possibilities to spread. The downside is the deletion of certain correct solutions (as is the case for trivial Q1, which produces lower means). Thanks to this, however, the optimal solution prevails and the incorrect ones tend to disappear from the system. Successive large-scale deletions can negatively affect mean scores, as can be seen in Q1 and Q3; in the remainder of cases, however, this process improves the system.

Finally, we arrive at Phase 6, where there are no deletions and students can only copy from Top10. Q1 is stable in the high score section, and all other questions significantly improve, exhibiting positive slopes.

We observe that at the end of the process, a sole collective solution has been achieved, but each user maintains their own solution. Our platform has been designed to spread ideas globally; in other words, the promotion of the most popular ideas, interaction, etc., makes these ideas reach practically all the users on the platform and attain a high density in the lattice. From this point of view, and once more recurring to the physical simile, we could say that these solutions percolate.

### Dynamics of fake responses

As described in the Methods Section, the mathematics examination contained a special question designed to detect copies from incorrect solutions obtained from the Internet. Allowing ourselves a certain abuse of language, we might call this the injection of “fake news” in Q5. The purpose is to check whether the system and its dynamics are able to filter out these responses or not. Clearly, to be able to confide in the experiment’s conclusions, it is very important to verify that the system can internally filter such bogus Items. To achieve this, we add an additional grade scoring Q5 as if the fake answer was the correct one: that is to say, we assign 10 points when the response is “84” (fake) and 0 otherwise. The evolution of Q5T (true) and Q5F (fake) in Figure 4 is then indeed remarkable. Up to the middle of phase 4, Q5F is above Q5T; that is to say, a larger number of students chose the fake solution. Only at the middle of phase 4, Q5T starts to grow, and the two scores cross. Q5T is selected by a large number of students in Phase 5, when deletions increase and Top10 becomes visible. We thus conclude that the possibility of viewing the best solution, when both responses are present, reinforces the choice of the correct one by a large number of participants.

We conclude that when students can only see a reduced number of neighbors, even if they have different solutions (the correct one, for instance), they do not tend to adopt other answers. Only from the moment when those differences are made visible in the Top10, the argument from authority becomes capable of leading most students to change their opinion.

In both MD and MA, participants injected a considerable number of meaningless responses, offensive ones, responses out of context, etc., which we call troll answers: reflecting, for instance, tendencies from social networks, or the espousal of a political program. These troll answers spread in the system, generally with low relevance, albeit with some exceptions on the Top 3, but are never present in final best solutions, as we explain in the following sections.

Nonetheless, the presence of “fake news” or Trolls is significant. For Q5 in MA, in Phase 5, the correct (and finally best) solution was selected by the 20% of participants, whereby the same percentage chose the fake one. By the end, 52% of the students had the correct answer, and only 18% proposed the fake one, which still represents a significant number of students. We thus see how the final phases where students are exposed to the Top10 solutions are crucial in order for them to choose the correct answers.

### Evolution of the best solutions

**FIGURE 5:**
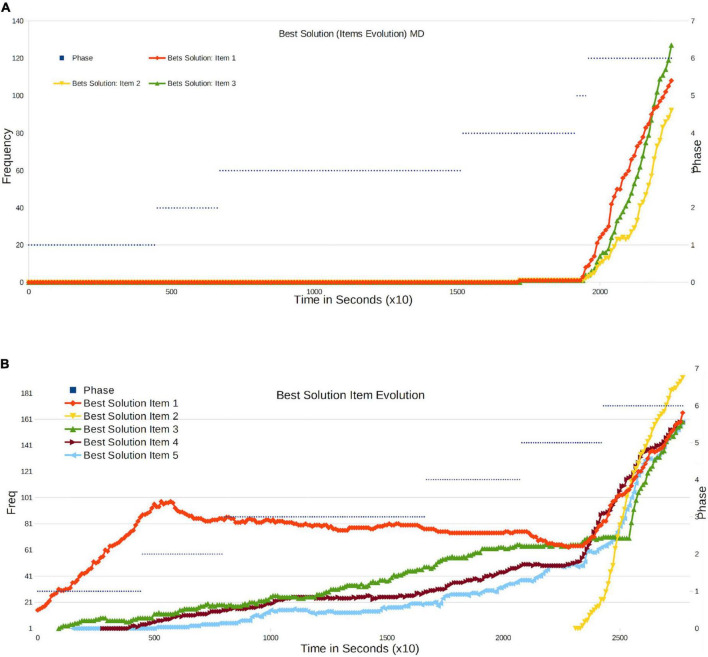
**(A)** Frequency evolution of the items in the Best Final Response in MD (three items). **(B)** Frequency evolution of the items in the Best Final Response in MA (five items).

We can also consider the evolution of the best solutions of the system from the vantage point of a grading system. Corresponding results are shown in Figures 5A,B. For each solution [3 (MD) or 5(MA) Questions], we rank them globally according to the sum of their Items frequencies. Note that with this definition, not necessarily all the best items are in the best solution.

• For MD, we observe that all the Items of the Best Solutions are generated at the end of phase 4, at the very moment when overwriting becomes stronger.
• In MA, for the Q1 control question we observe a larger slope at the beginning, indicating that the majority of students are working on the examination seriously. From the middle of Phase 2, noise from the system produces a negative slope, but the system ultimately returns to the correct solution with pronounced growth in Phase 5. The quality of responses to all questions evolves positively, with increasing frequency when a greater number of students copy or create correct solutions. It is true that the platform does introduce noise, and that global information lies below a controlled number that is lower at the end. The most remarkable fact is that all non-trivial questions (Q2-Q5) exhibit a remarkable change in behavior in the middle of Phase 5, when the possibility of overwriting is introduced and the students can view Top10 solutions. In fact, the final response to Q2 appears just at this moment and grows considerably from that point on. By the time the process has concluded, the Best Solution (Top1) is the correct one for all questions, from Q1 to Q5 (Score 10/10), with a very high number of students opting for the best solution. The mean score lies below the maximum (10) because of the presence of other lower-frequency solutions. In any case, the most remarkable finding is that the most frequent solution is the correct one for all Items (Score: 10/10).

From this, we can conclude that the influence of the group’s size on its performance is clear: we can see how in all non-trivial questions, the overall performance increases with the phase, which implies that the interaction between an increasing number of students (since their neighbors are interacting at the same time with others) has a positive effect on the group’s performance, with a linear dependence. Notice that the change in overall performance cannot be attributed to the increment of time, since the activity has a parallel increment in the number of copies (refer to Figure 3); thus, it must be related to the interaction. Nonetheless, the strongest improvement in performance appears at the moment when the students are allowed access to the group’s best average results, as presented in the Top10 solutions. In this sense, we can claim that the moment from which students are given the chance to interact with all other students (through the set of average answers) is when improvement becomes most remarkable. This can be due to the group’s size, but also to the weight of the group as such on the opinion of its individual members. In conclusion, we ascertain a remarkable dependence of performance on group size and a very significant dependence on average opinion.

## Conclusion

This study’s aim has been to analyze the interaction dynamics in an experiment conceived and carried out on the Thinkhub platform. Designed to foster collective intelligence, Thinkhub tackles the usual problems of interactions in large groups by applying two important mechanisms: an interaction model that allows for the diffusion of ideas generated in the course of the experiment, and a moderation model based on artificial intelligence, which applies a suppression mechanism to less popular answers. The experiment shows that the system we have created is capable of enhancing collaborative efforts in a group and, without external intervention, of leading the group to produce a high-quality answer to a difficult task. We have proven how interaction within small groups that grow in size over time is able to produce a percolation dynamic for the ideas created by the individuals, which spread across the group (Stauffer and Aharony, 1992). This process exhibits an approximately linear increment that runs in parallel with group size, both in terms of the generation of ideas to solve the task and in terms of the quality of the answers. Finally, the most significant portion of the process is associated with a final phase where the members of the group are provided with the most popular answers to the task, which in all cases of our experiment include the best answer. The final most popular answer corresponds to the best one in all cases. We presume that the reason why this last phase turns out to be crucial is because the individual is much more receptive to the influence of the group as an entity than to that of other individuals as such. This is why the student accepts an answer as true, because they know that it comes from the group and a majority of members have validated it.

Apart from this, we encountered the problem that the system was sensitive to potential disturbances generated by certain members who were able to create troll answers, which also spread across the group. Nonetheless, the system was able to filter out those disturbances and boost the correct answers to make them invariably prevail, in two global tasks of thoroughly different nature, and in all questions featured in each task. The appearance of this phenomenon may relate our case to situations arising in large group interactions, such as the appearance of fake news (Scheibenzuber et al., 2021). The Thinkhub platform includes the option of adding troll users with answers created by the researchers themselves, to study the dynamics generated by such answers and hence to experimentally study the conditions which contribute to the spread of fake news. This context can obviously not be compared with the evolution of fake news in real life, as it does not take into account the motivation or emotional dimensions of real users who create it, nor the conditions that permit us to evaluate its impact (Colliander, 2019; Sethi et al., 2019; Yang and Tian, 2021). Nevertheless, this methodology can serve as a complement to existing experimental studies of surveys or case studies (Jang et al., 2018; Bastick, 2021).

As we have already stated in the introduction, it is possible that the appearance of collective intelligence may depend on contextual factors (Almaatouq et al., 2020; Sulik et al., 2022). In this sense, with this work, we would have revealed some of the conditions that could make this CI emerge in large groups. On the one hand, working conditions in small groups guarantee the activity of the participants in them and the dissemination of information. The previous study developed by Navajas et al. (2018) tested a similar model where it began with a phase of work in small groups and with a second phase of expansion of information and search for feedback in large groups, a process that would also be included in the last phases of our study. Both in ours and in the already mentioned study by Navajas et al. (2018), the model is oriented toward the search for consensus, directing the system to obtain this response. Thus, the effectiveness of our system is guaranteed by the feasibility of using artificial intelligence mechanisms to obtain this response.

Another second contextual aspect to take into account to explain the results obtained could be given by assuming that exposing the participants to the information generated supposes creating a context of influence or social learning. From this idea, we assume that learning really takes place during the process and that, furthermore, as we have mentioned, the final phases in which the system selects the answers entail a process of validation of those answers. This would justify considering social influence as an important condition in the contexts of large groups (Lorenz et al., 2011; Fontanari, 2018; Kabo, 2018; Toyokawa and Gaissmaier, 2022).

However, we know that these mechanisms of social influence can generate negative results and the expansion of low-quality ideas (Sulik et al., 2022; Toyokawa and Gaissmaier, 2022), but we cannot affirm that this interaction model can protect this type of process. We have verified that the model can reduce the spread of trolls and fake news, but we are not sure that this can be the case in all situations. To do that, more work would be necessary, since there are numerous contextual key factors, as the motivation of the participants.

A field where it could be interesting to address the studies of this type could be in regulated learning situations, such as those considered in the contexts of Computer Supported Collaborative Learning (CSCL) where large groups of students interact to learn with well-defined performance motivations (Castellanos et al., 2017; Scheffel et al., 2017; Holtz et al., 2018).

There are other contextual variables that we have not investigated in this study and that could be relevant in future analyses, such as the personal characteristics of the participants, already taken into account in other studies (Hjertø and Paulsen, 2016; Aggarwal et al., 2019; Woolley and Aggarwal, 2020). These characteristics may determine more or less conservative action strategies of the participants Toyokawa and Gaissmaier (2022) and generate other well-known negative effects such as the herd effect that may cause low activity in the interaction process (Mao et al., 2016; Toyokawa et al., 2019; Sulik et al., 2022). Having platforms like the one presented here, in which there is a record of activity in each phase, makes it possible to analyze the possible effects and search for alternatives, if necessary. These alternatives may include, for instance, non-random mechanisms for assigning participants to groups, which have been considered in other works (Almaatouq et al., 2020).

Our experiment has certain limitations. The most important one is that the interaction system is defined *a priori* and hence establishes the properties of the interaction during the experiment, such as the duration of each phase or the mechanism applied to suppress unpopular answers. Despite this limitation, the system is sufficiently flexible to allow the researchers to control many of the above-mentioned parameters; moreover, it includes interesting features such as the possibility of adding artificial answers *via* automatic troll-users. A second limitation of our study is associated with the evaluation of the results of the moral dilemma. Indeed, our approach to grade the different answers by resorting to Kohlberg’s stages (Kohlberg, 1976, 1989) may be insufficient due to the participants’ homogeneity in terms of age and may not be capable of discriminating among the complexity of the given answers. This issue exemplifies the complexity of analyzing large amounts of data from a semantic perspective. This framework could nevertheless represent a promising alternative for the analysis of large quantities of data, such as those produced on social networks (Sethi et al., 2017; Lozano-Blasco et al., 2021).

## Data availability statement

The raw data supporting the conclusions of this article will be made available by the authors, without undue reservation.

## Ethics statement

The studies involving human participants were reviewed and approved by CEIC Aragón (CEICA). The patients/participants provided their written informed consent to participate in this study.

## Author contributions

SO, JC-G, and AT: generation of ideas, literature review, conceptual framework, methodology, data collection, analysis of data, conclusion and discussion, first writing, second writing review, and funding. JC-E: generation of ideas, methodology, data collection, analysis of data, conclusion and discussion, and first writing. AC-S: generation of ideas, methodology, data collection, conclusion, and discussion. PB: literature review, conceptual framework, analysis of data, conclusion, and discussion. AR: generation of ideas, conceptual framework, methodology, data collection, analysis of data, conclusion, and discussion. PR: generation of ideas, methodology, analysis of data, conclusion, and discussion. All authors contributed to the article and approved the submitted version.

## Footnotes

1
https://bifi.es

2
https://www.kampal.com/

3
https://ic.kampal.com/projects
