# Corrigendum: Evolutionary emergence of collective intelligence in large groups of students

**DOI:** 10.3389/fpsyg.2023.1168563

**Published:** 2023-05-03

**Authors:** Santos Orejudo, Jacobo Cano-Escoriaza, Ana Belén Cebollero-Salinas, Pablo Bautista, Jesús Clemente-Gallardo, Alejandro Rivero, Pilar Rivero, Alfonso Tarancón

**Affiliations:** ^1^Department of Psychology and Sociology, University of Zaragoza, Zaragoza, Spain; ^2^Department of Sciences of Education, University of Zaragoza, Zaragoza, Spain; ^3^Department of Theoretical Physics, University of Zaragoza, Zaragoza, Spain; ^4^Institute for Biocomputation and Physics of Complex Systems, University of Zaragoza, Zaragoza, Spain; ^5^Kampal Data Solutions, Zaragoza, Spain; ^6^Department of Specific Didactics, University of Zaragoza, Zaragoza, Spain

**Keywords:** collective intelligence, social experiment, web platform, mathematics test, moral reasoning

In the published article, there was an error in the Funding statement. Full information on the funding was not provided. The correct Funding statement appears below.

The authors apologize for this error and state that this does not change the scientific conclusions of the article in any way. The original article has been updated.

